# Supplementary material for: Factors influencing secondary school students’ nutrition, mindfulness, and academic performance in Nan Province, Thailand
Source: PLoS One. 2025 Jan 14;20(1):e0308882. doi: 10.1371/journal.pone.0308882 (PMC11731758; doi:10.1371/journal.pone.0308882)
Supplement: S1 Table — (DOCX) [file pone.0308882.s001.docx]

**S1 Table.** **Academic Performance of the Participants**

| **Grade point average (GPA); n=350** | **Number (%)** |
| --- | --- |
| Grade A | 5(1.4) |
| Grade B | 173(49.4) |
| Grade C | 129(36.9) |
| Grade D | 43(12.3) |
