# Supplementary material for: Factors influencing secondary school students’ nutrition, mindfulness, and academic performance in Nan Province, Thailand
Source: PLoS One. 2025 Jan 14;20(1):e0308882. doi: 10.1371/journal.pone.0308882 (PMC11731758; doi:10.1371/journal.pone.0308882)
Supplement: S2 Table — Anthropometric assessments S2.1 Table. Body Mass Index, S2.2 Table. Waist circumference ratio and S2.3 Table. BMI by GPA. (DOCX) [file pone.0308882.s002.docx]

**S2 Table. Anthropometric Assessments**

**S2.1 Table.** Body Mass Index

| **Gender** | **Body Mass Index (BMI; kg/m^2^): n(%)** | | | |
| --- | --- | --- | --- | --- |
|  | **Under weight** | **Normal weight** | **Over weight** | **Obesity** |
| Male | 26(20.8) | 70(56.0) | 16(12.0) | 13(9.6) |
| Female | 30(13.3) | 151(67.1) | 24(10.7) | 20(8.9) |
| Total | 56(16.0) | 221(63.1) | 40(11.4) | 33(9.4) |

**S2.2** **Table. Waist circumference ratio**

| **Gender** | **Waist circumference ratio group: n(%)** | |
| --- | --- | --- |
|  | Normal | Risk |
| Male | 113(35.5) | 12(37.5) |
| Female | 205(64.5) | 20(62.5) |
| Total | 318(90.86) | 32(9.14) |

**S2.3 Table. BMI by GPA**

|  | | **GPA** n(%) | | | | | |
| --- | --- | --- | --- | --- | --- | --- | --- |
|  |  | Excellent | Good | | | Fair | Poor |
| **BMI** | Underweight | 0(0.0) | | 25(44.64) | 25(44.64) | | 6(10.71) |
|  | Normal | 4(1.81) | | 126(57.01) | 66(29.86) | | 25(11.31) |
|  | Overweight | 1(2.50) | | 16(40.00) | 17(42.50) | | 6(15.00) |
|  | Obese | 0(0.0) | | 6(18.18) | 21(63.64) | | 6(18.18) |
