# Supplementary material for: Factors influencing secondary school students’ nutrition, mindfulness, and academic performance in Nan Province, Thailand
Source: PLoS One. 2025 Jan 14;20(1):e0308882. doi: 10.1371/journal.pone.0308882 (PMC11731758; doi:10.1371/journal.pone.0308882)
Supplement: S3 Table — (DOCX) [file pone.0308882.s003.docx]

**S3 Table. Knowledge, Attitude score level About Nutrition and** Classroom Environment

| **Gender** | **Knowledge score level About Nutrition;** n(%) | | | **Attitude score level About Nutrition;** n(%) | | **Attitude score level About** Classroom Environment**;** n(%) | |
| --- | --- | --- | --- | --- | --- | --- | --- |
|  | Good | Fair | Poor | **Good** | **Fair** | **Good** | **Fair** |
| **Male** | 17(13.60) | 43(34.40) | 65(52.00) | 58(46.40) | 67(53.60) | 17(12.80) | 43(87.20) |
| **Female** | 72(32.00) | 82(36.44) | 71(31.56) | 134(59.56) | 91(40.44) | 72(19.56) | 82(80.44) |
