# Supplementary material for: Factors influencing secondary school students’ nutrition, mindfulness, and academic performance in Nan Province, Thailand
Source: PLoS One. 2025 Jan 14;20(1):e0308882. doi: 10.1371/journal.pone.0308882 (PMC11731758; doi:10.1371/journal.pone.0308882)
Supplement: S4 Table — (DOCX) [file pone.0308882.s004.docx]

**S4 Table. Eating behavior of the participants**

| **Eating behavior** | **Number (%)** |  |
| --- | --- | --- |
| **Number of meals per day** |  |  |
| 1 meal | 114 (32.5) |  |
| 2 meals | 85(24.2) |  |
| 3 meals | 151(43.1) |  |
| **Skip meal** |  |  |
| No | 72(20.6) |  |
| Yes | 278(79.4) |  |
| **How often skip** |  |  |
| Every day | 22(7.9) |  |
| 1/2times per week | 158(56.8) |  |
| 3/4times per week | 68(24.5) |  |
| 5times per week | 21(7.6) |  |
| 6times per week | 9(3.2) |  |
| **Why skip** |  | |
| No enough time | 127(45.7) | |
| Keep weight | 28(10.1) | |
| Habit | 34(12.2) | |
| Don’t like food | 89(32.0) | |
| **Consuming junk food (like potato chips, cookies, cakes)** |  | |
| No | 181 (51.7) | |
| Yes | 169 (48.3) | |
| **How often do you eat snack** |  | |
| Once per day | 73(43.7) | |
| Twice per day | 63(37.0) | |
| 3 times per day | 22(12.8) | |
| More than 3 times per day | 11(6.5) | |
| **Why you take snacking** |  | |
| Hungry | 31(18.4) | |
| To eat as a meal | 12(7.1) | |
| Like them | 81(47.9) | |
| Habit | 45(26.6) | |
| **How many glasses(250ml) of drinking water per day** |  | |
| >8 glasses | 82(23.4) | |
| <8 glasses | 268(76.6) | |
| **Serving milk (220 ml) or yogurt per day** |  | |
| >220 ml | 287(82) | |
| <220 ml | 63(18) | |
