# Supplementary material for: Factors influencing secondary school students’ nutrition, mindfulness, and academic performance in Nan Province, Thailand
Source: PLoS One. 2025 Jan 14;20(1):e0308882. doi: 10.1371/journal.pone.0308882 (PMC11731758; doi:10.1371/journal.pone.0308882)
Supplement: Attachment — Submitted filename: Responds to reviewers.doc [file pone.0308882.s005.doc]

'Point-by-point response to editors and reviewers'

According to comments, we agreed and corrected with all the comments raised by the editors and reviewers. We would like to take this opportunity to express our sincere thanks to editor and reviewers who identified areas of our manuscript that needed corrections or modification. We would like also to thank you for allowing us to resubmit a revised copy of the manuscript.

Reviewer 1:

| Reviewer 1: Comments | **Author’s Response** |
| --- | --- |
| Reviewer #1: Thanks the authors for bringing such interesting study to the scientific community. I have forwarded my comments regarding the study as follows:  1. The authors have described the variables and definitions of the study in their manuscript. But it is not clear whether these are adapted or adopted. | Thank you for your comment. We've clarified our measures as follows:   1. Anthropometric measurements: Adopted from WHO standards (2007). 2. Nutritional knowledge: Adapted from Thailand's five food groups guidelines 3. Attitude About Food Intake: Self-developed using a five-point Likert scale. 4. Consuming Behavior: Self-developed to assess eating habits and food choices. 5. Perception About Environmental Factors: Self-developed using a five-point Likert scale. 6. Mindfulness Practice: Adopted the Child and Adolescent Mindfulness Measurement (CAMM) by Greco et al. (2011). 7. Academic performance measurement: Adopted the standard GPA categorization used in the Thai education system.   Self-developed measures were pre-tested. We'll revise our manuscript to clearly indicate these origins with appropriate citations.  **See in Page 4 lines 6-8: lines 134-175.** |
| 2. The manuscript lacks in defining some variables for example; Consuming Behavior, Perception about Environmental Factors and Mindfulness Practice. | Thank you for your feedback. We've added clearer definitions for some variables:   1. Consuming Behavior: Assesses eating habits, meal frequency, and food choices. 2. Perception about Environmental Factors: Measures opinions on school environment (air, light, temperature, noise, space). 3. Mindfulness Practice: Evaluates present-moment awareness and non-judgmental attitudes using CAMM.   These definitions are now included in our methodology section. We appreciate your help in improving our manuscript's clarity.  **See in Page 7: lines 152-176.** |
| 3. The study also lacks in showing how the academic performance of the study participants are scored and measured? | Thank you for your comment regarding the scoring and measurement of academic performance. We have addressed this concern in our revised manuscript.  We have added a detailed description of how academic performance was assessed:  “Academic performance was measured using the previous year's grades (2022-2023) from Bo Kluea School's information system. We adopted the standard Thai education system GPA categorization, ranging from 0 to 4. Categories include Excellent (4.00, Grade A) to Fail (0-0.99, Grade F). This standardized system provided a consistent, objective measure of academic performance across all participants.”  This addition clarifies the source of academic data, the grading scale used, and how it was applied in our study. We believe this addresses your concern about the scoring and measurement of academic performance.  **See in Page 6: lines 134-140.** |
| 4. Information regarding sample (sample size and sampling) should be clearly stated before the result of the study. | Thank you for your comment regarding the sample size and sampling information. We have addressed this concern by adding a new section in our Materials and Methods:  “Study Population and Sampling  The study targeted all students from grades 8-11 at Bo Kluea School, employing a total population sampling approach. The total number of eligible students in these grades was 420. This comprehensive sampling method aimed to include all students who met the inclusion criteria and did not fall under the exclusion criteria. The approach was chosen to ensure a representation of student population of the school, while adhering to ethical considerations. Students were recruited through school announcements. The final sample size was determined after applying the inclusion and exclusion criteria, with the actual number of participants reported in the Results section”.  We believe this addition provides clear information about our target population  **See Page 5: line 97-104** |
| 5. If the authors have checked the validity and reliability of the tool, there is the need to display internal validity and test-retest reliability, with the Cronbach’s α. | Thank you for your valuable comment regarding the validity and reliability of our tools. We acknowledge the importance of these aspects and appreciate the opportunity to address them:   1. Pre-testing: We conducted a pre-testing phase with a small group of approximately 5 individuals. This primarily assessed the comprehensibility of our questionnaires and ensured that the questions were clear to the target audience. However, we recognize that this was not sufficient for establishing formal validity and reliability due to the small sample size. 2. Tool development: The nutrition knowledge, attitude, and behavior sections were developed based on Thailand's five food groups guidelines and our team's extensive experience (over 10 years) in nutrition research. While this expertise informed the questionnaire design, we acknowledge that formal validity testing was not conducted prior to the study. 3. CAMM scale: For the mindfulness practice section, we adopted the validated Child and Adolescent Mindfulness Measurement (CAMM) developed by Greco et al. (2011), which reported a Cronbach's α of 0.81 in their original study. 4. Post-hoc reliability: After completing the study, we calculated Cronbach's α for our questionnaire sections using the full sample (n=350) to estimate reliability. The results are as follows:    - Knowledge of Nutrition: α = 0.646    - Attitude About Food Intake: α = 0.691    - Perception About Environmental Factors: α = 0.705    - Mindfulness Practice (CAMM): α = 0.875   We have added the following limitation to our discussion section to ensure transparency in our reporting:  'A notable limitation is the lack of comprehensive validity and reliability testing of our questionnaires prior to the main study. Our pre-testing phase, conducted with a small group of approximately 5 individuals, was primarily to assess the comprehensibility of our questionnaires. We did not conduct formal validity checks or calculate Cronbach's α before the main study due to the small pre-test sample size and time constraints. For the CAMM scale, we relied on the previously reported psychometric properties. Post-hoc reliability analysis was conducted on the full sample to provide estimates of internal consistency.'  We appreciate your insight and acknowledge that more rigorous validity and reliability checks would strengthen our study. This feedback is valuable for improving our research methods in future studies  **See Page 14-15: line 319-325** |
| 6. Do the authors display the crude and adjusted odds ratio in one table for the better examinations of the association between outcome variable and the predictor variables? | We presented crude odds ratios for all variables in Tables 2 and 3 to provide a comprehensive view of initial associations. We then included only statistically significant predictors in our adjusted odds ratio tables (Tables 4 and 5) to focus on the most impactful factors after controlling for other variables. This approach enhances clarity, avoids overfitting, and aligns with common practices in epidemiological research. It allows readers to quickly identify the key factors influencing BMI and academic performance. While we recognize that showing both crude and adjusted odds ratios for all variables could offer additional insights, this would significantly increase the amount of information presented |

Reviewer 2:

| Reviewer 1: Comments | **Author’s Response** |
| --- | --- |
| Reviewer #2: It is an honour to review the manuscript. This is an interesting study on “Factors Influencing Secondary School 1 Students' Nutrition, Mindfulness, and 2 Academic Performance in Nan Province, Thailand”. My comments are appended.  Abstract: Abstract is too long, it is better to shorten. | **We revised and shorten the abstract as recommended.** |
| Introduction: Introduction has been written in right direction. However, in my opinion it is better to put some background information or research from Thailand to draw rationale or knowledge gap. | We incorporated relevant background information and research from Thailand to better establish the rationale and highlight the knowledge gap **as recommended following**  “In Thailand, the education system faces several challenges that impact academic performance. Despite government efforts to increase education spending, the system has suffered from chronic underfunding for decades, with lower expenditure compared to other countries [5]. This underfunding has led to inadequate educational resources, particularly in rural areas. Thailand also experiences one of the highest levels of educational inequality globally, with significant disparities in access to education and academic outcomes between rural and urban areas, as well as among different socioeconomic groups [6]. The COVID-19 pandemic has further exacerbated these issues, forcing many schools to switch to online learning without sufficient resources or training [7].”  **See in Page 2-3: lines 46-54** |
| Methods: Methods has been written in right direction. | We're pleased to hear these sections meet your expectations. |
| Results: Results has been written in right direction. | **Thank you** |
| Discussion: Discussion has been written in right direction. The author needs to put some limitations and strength of this study. | The limitations and strengths of our study are added following “Despite these significant findings, this study has several limitations. As it was conducted in a single school in Nan province, the results may not reflect to all Thai students or schools in different regions. The cross-sectional design limits our ability to establish causal relationships between the examined factors and academic performance. Additionally, self-reported data on nutrition and mindfulness practices may be subject to recall bias or social desirability bias. However, the study also has notable strengths. It provides a comprehensive examination of multiple factors influencing academic performance, including nutrition, environmental factors, and mindfulness, which is relatively rare in the Thai context. The large sample size (350 students) enhances the statistical power of the findings. Furthermore, the use of both subjective (questionnaires) and objective (measurements, academic records) data collection methods strengthens the validity of the results.”  **See Page 14-15: line 315-322**  We believe these changes will significantly improve our manuscript. Thank you again for your insightful review. |
